# Glomerulus-Specific Inhomogeneity of the Basal Activity Map in the Olfactory Bulb

**DOI:** 10.3390/ijms27083684

**Published:** 2026-04-21

**Authors:** Stefan Fink, Natalie Fomin-Thunemann, Farzin Kamari, Yury Kovalchuk, Olga Garaschuk

**Affiliations:** 1 Institute of Physiology, Department of Neurophysiology, Eberhard Karls University of Tübingen, 72074 Tübingen, Germany; stefan.fink@medizin.uni-tuebingen.de (S.F.); farzin.kamari@uni-tuebingen.de (F.K.);; 2 Department of Otolaryngology, Head and Neck Surgery, Division Hearing, Cognition and Tinnitus, Tübingen Hearing Research Centre, 72076 Tübingen, Germany; 3 Department of Biomedical Engineering, Boston University, Boston, MA 02446, USA

**Keywords:** olfactory signal processing, in vivo calcium imaging, first stage of olfactory sensation, awake recordings

## Abstract

Glomeruli are signal-processing units of the olfactory bulb (OB) that play a key role in many OB computations, including contrast enhancement, gain control, and odorant-selective habituation. In awake mice, we unveil an extremely stable, inhomogeneous map of basal glomerulus-specific activity that serves as the background against which olfactory signal processing occurs. This activity is strongly driven by centrifugal cholinergic inputs; endogenous and airflow-evoked spiking of olfactory sensory neurons; and, to a minor extent, by the odor environment. Moreover, it is brain-state dependent and suppressed under various forms of anesthesia, and is therefore likely attenuated during sleep. These results reveal an important layer in the OB signal-processing network, likely increasing the system’s variance and dynamic range via glomerulus-specific functional inhomogeneity.

## 1. Introduction

The olfactory bulb glomeruli are fundamental units representing the first stage of sensory processing in olfaction. In mice, a typical glomerulus receives inputs from ~1000 olfactory sensory neurons (OSNs), all expressing the same type of odor receptor (OR). Within the glomerulus, the OSN axons synapse on primary dendrites of 20–50 mitral/tufted (M/T) cells and hundreds of juxtaglomerular interneurons [1,2]. Experimental and computational modeling data suggest that intraglomerular computations play a key role in the contrast enhancement, gain control, odorant-selective habituation, and odorant-specific suppression of M/T cell outputs [3,4,5]. Because the chemoreceptive properties of different OSNs overlap such that each odor activates many ORs to a greater or lesser extent, it is largely assumed that it is the combinatorial pattern across the set of corresponding odor-activated glomeruli that forms the basis for odor perception and identification. Accordingly, perceptually similar odors activate a correspondingly greater number of common ORs/glomeruli, thus evoking highly overlapping primary odor representations, which are then decorrelated by the OB circuitry [6,7]. This logic, however, implicitly assumes that odor-evoked OSN signals are projected onto a layer of “resting” glomeruli, with some nonspecific background noise [8,9,10,11]. This noise may comprise the spontaneous activity of OSNs, known to fire at frequencies between 0 and 7 Hz [12,13,14,15,16], as well as that of spontaneously active juxtaglomerular interneurons and M/T cells [17,18], randomly distributed in the glomerular layer in a salt-and-pepper fashion.

Here, using in vivo two-photon Ca^2+^ imaging in awake, head-restrained mice with neuronal expression of the ratiometric Förster resonance energy transfer (FRET)-based Ca^2+^ indicator Twitch-2B under a chronic cranial window, we challenge the above view by showing that the majority of glomeruli are not silent. Rather, the “resting state” of the glomerular layer of the OB is characterized by highly inhomogeneous, stable, and glomerulus-specific patterns of endogenous activity. The preprint of this study was posted on bioRxiv [19].

## 2. Results

### 2.1. Inhomogeneity of the Glomerular Map in Resting Awake Animals

**Figure 1:**
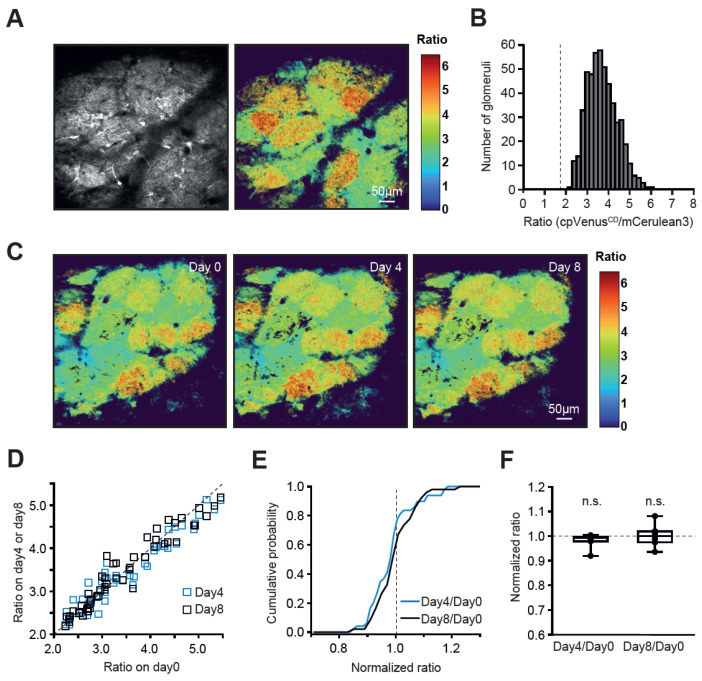
**Inhomogeneity and stability of glomerular basal Twitch-2B ratios in awake mice.** (**A**) Left: Average-intensity projection image of the dorsal surface of the OB (40–58 µm depth), labeled with the Ca^2+^-indicator Twitch-2B. Right: Corresponding average projection map of the color-coded cpVenus^CD^/mCerulean3 ratios, hereafter referred to as “color map”. Note that each glomerulus appears on the map in a distinct, largely homogeneous color. (**B**) Histogram illustrating the distribution of basal Twitch-2B ratios of individual glomeruli (n = 506 glomeruli, 23 mice). A broken vertical line marks the spiking threshold (see Figure S2C). (**C**) Sample glomerular color maps of an OB region taken at three different time points (Day 0, Day 4, and Day 8). (**D**) Scatter plot showing basal glomerular Twitch-2B ratios recorded at Days 4 and 8 plotted against the corresponding ratios recorded at Day 0 (n = 49 glomeruli, 7 mice). Diagonal broken line: Unity line. (**E**) Cumulative probability plot displaying the distribution of normalized basal glomerular Twitch-2B ratios in the data set shown in (**D**). (**F**) Box plot showing the medians (per mouse) of normalized ratios, which are not significantly (n.s.) different between days (Friedman’s test *p* = 0.18, χ^2^(2) = 3.4).

All experiments were conducted in awake, head-restrained mice that were extensively trained and habituated to the imaging setup before recordings. Adenovirus injections into the glomerular layer of the OB enabled the labeling of OSN axons, as well as periglomerular and mitral/tufted cells, with the FRET-based Ca^2+^ indicator Twitch-2B (see Section 4 for details). As shown in Figure 1A, structures with different background-corrected ratios of acceptor to donor fluorophore fluorescence (cpVenus^CD^/mCerulean3, hereafter referred to as Twitch-2B ratios) coincided in shape and size with individual glomeruli, visible in the fluorescence image of the glomerular layer (left panel). The mean basal ratios recorded from different glomeruli ranged from 2 to 6 (Figure 1B, n = 506 glomeruli in 23 mice), well within the dynamic range of the Twitch-2B Ca^2+^ sensor, ranging from 1.3 (see below) to 8.5 [20]. The observed inhomogeneous maps were surprisingly stable across different experimental days (Figure 1C–F and Figure S1).

To test whether the differences in the basal Twitch-2B ratios among individual glomeruli reflect differences in the action potential firing of underlying network elements, we applied the sodium channel blocker tetrodotoxin (TTX, 5 µM) on top of the cranial window with a slit [21]. Topical TTX application strongly reduced the basal Twitch-2B ratios, completely wiping out map inhomogeneity (Figure S2). Note that under TTX, all basal Twitch-2B ratios were below 1.8 (broken line in Figure S2C). Therefore, glomeruli with basal ratios ≤ 1.8 were considered non-active (i.e., comprising non-spiking cells), whereas the ratios ≥ 2 were considered to reflect the spiking of contributing neuronal elements. This conclusion was further substantiated by recordings at a higher sampling rate (~8 Hz), which revealed ongoing fluctuations of the intracellular Ca^2+^ levels in glomeruli with high Twitch-2B ratios (Figure S3). Surprisingly, neuronal elements within a single glomerulus had rather similar colors (i.e., Twitch-2B ratios) and, thus, spiking frequencies, underscoring the functional unity of individual glomeruli [18]. According to our cell soma-based in situ calibration [20], ratio values of 2–3 correspond to mean neuronal spiking frequencies of 0–5 Hz, whereas ratio values of 6 correspond to those of 20 Hz. Notably, in awake mice, we did not observe any non-spiking glomeruli (Figure 1B).

### 2.2. Underlying Cellular and Molecular Mechanisms

**Figure 2:**
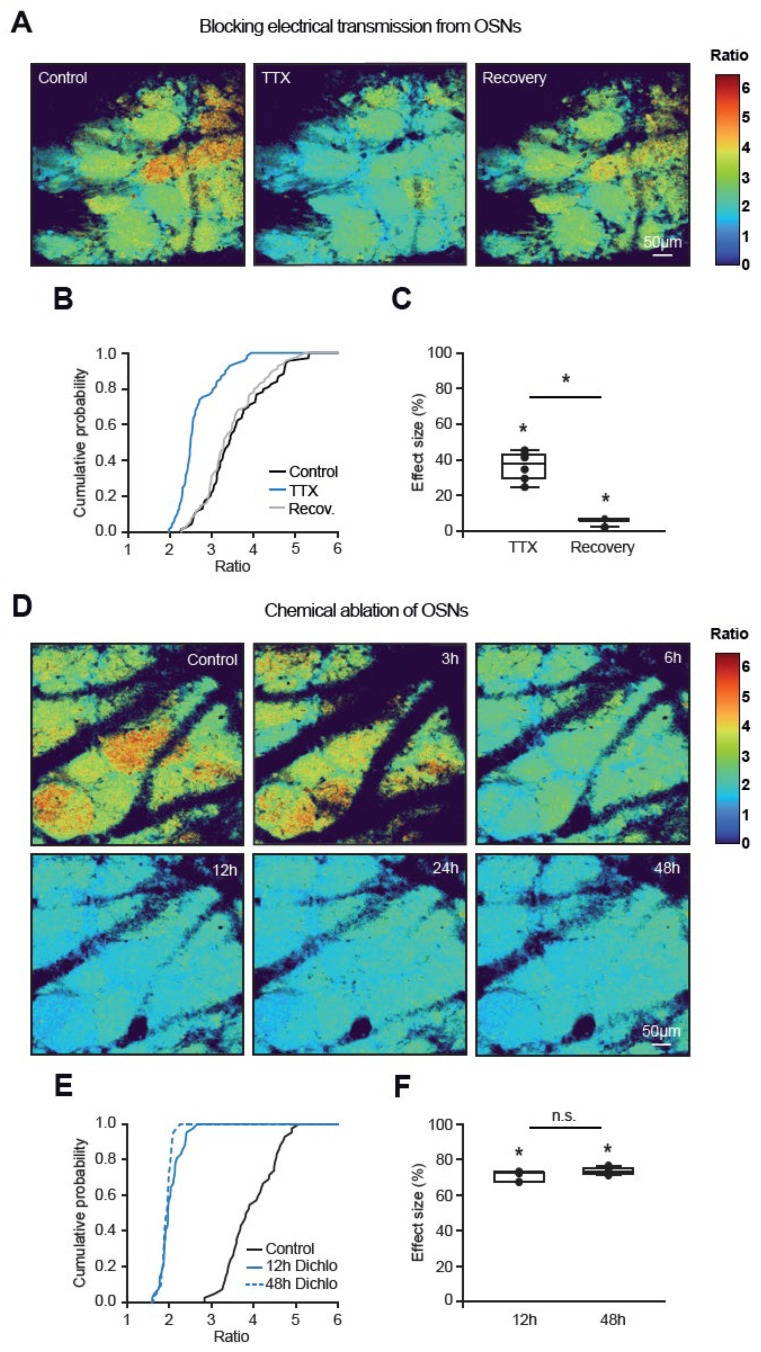
**Activity maps depend on action potential firing of intact OSNs.** (**A**) Color maps illustrating a reduction in basal Twitch-2B ratios upon intranasal application of 50 µM TTX and their partial recovery 24 h after application. (**B**) Cumulative probability plot showing the distributions of basal Twitch-2B ratios before (black), during (blue), and 24 h after (gray) TTX application (n = 70 glomeruli, 6 mice). (**C**) Box plot displaying the median (per mouse) effect sizes (calculated as described in Section 4; n = 6 mice) of TTX blockade and recovery. TTX and recovery effect sizes significantly differ from zero (Friedman’s test *p* = 2.5 × 10^−3^, χ^2^(2) = 12, post hoc Conover test with Bonferroni correction: *p* < 2.2 × 10^−16^ for all comparisons). (**D**) Color maps illustrating the gradual decrease in basal glomerular Twitch-2B ratios within hours after i.p. injection of dichlobenil (50 µg/g body weight). (**E**) Cumulative probability plot showing distributions of basal ratios before (black) and 12 (blue) and 48 h (broken blue line) after dichlobenil injection (n = 42 glomeruli, 6 mice). (**F**) Box plot showing the effect sizes of induced changes 12 and 48 h after dichlobenil injection (Friedman’s test, *p* = 5.7 × 10^−3^ χ^2^(2) = 10.3; post hoc Conover test with Bonferroni correction: *p* = 1.7 × 10^−3^ for 12 h, *p* = 4.5 × 10^−5^ for 48 h, and *p* = 5.3 × 10^−2^ for comparison between 12 and 48 h). * *p* < 0.05, n.s. = not significant.

**Figure 3:**
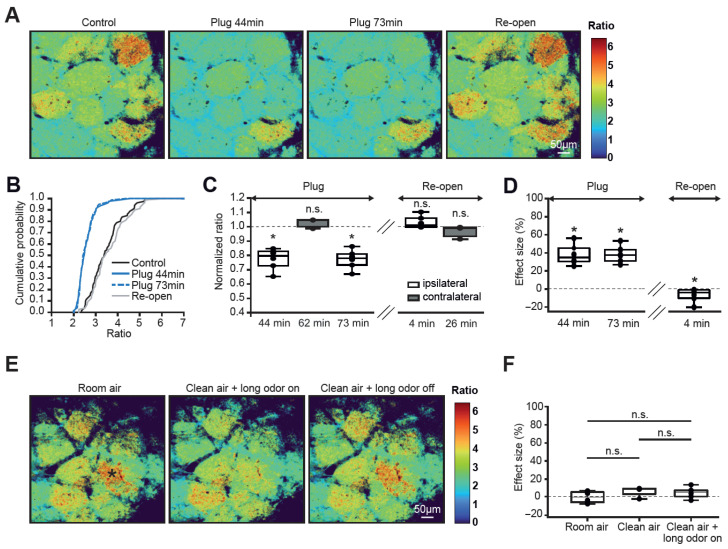
**Activity maps are affected by nasal airflow.** (**A**) Color maps illustrating basal Twitch-2B ratios before (Control), during (Plug), and after (Re-open) reversible plugging of one nostril. (**B**) Cumulative probability plot showing distributions of basal ratios before (black), during (blue), and after (gray) the nostril plugging (n = 80 glomeruli, 6 mice). (**C**) Box plot showing the medians (per mouse) of normalized (to immediately preceding control) basal ratios of glomeruli ipsi- (white, n = 6 mice) and contralateral (gray, n = 3 mice) to the plugged nostril. Time points on the *x*-axis indicate the duration of nostril plugging (Plug) or time after plug removal (Re-open; mixed-model ANOVA with post hoc Tukey test, *p* < 10^−4^ for 44 and 73 min and *p* ≥ 0.66 for all other comparisons). (**D**) Box plot showing effect sizes of ratio changes (Friedman’s test, *p* = 1.03 × 10^−3^ χ^2^(3) = 16.2, post hoc Conover test with Bonferroni correction: compared to zero effect size *p* = 2.1 × 10^−4^ for both 44 and 73 min, and *p* = 9 × 10^−3^ for Re-open). (**E**) Color maps illustrating basal Twitch-2B ratios in room air, during continuous application of isoamyl acetate (IAA, 25–29 min after odor on, 0.18% s.v.) and 10–14 min after the application (odor off). (**F**) Box plot showing effect sizes of ratio changes measured during the 30-minute-long period in room air, clean air, and clean air + long odor on (Kruskal–Wallis test, *p* = 0.13; n = 6, 4, 6 mice, respectively). * *p* < 0.05, n.s = not significant.

Next, we focused on mechanisms sustaining the glomerular map inhomogeneity in the resting state. To estimate the contribution of the OSNs, we applied 50 µM TTX in 10 µL PBS intranasally, re-examined the glomerular activity map 20–30 min after the TTX application (Figure 2A,B), and quantified the blocker’s effect size, i.e., the ratio change under test conditions normalized to its full dynamic range (Figure 2C, 38.02 ± 13.52, n = 69 glomeruli, 6 mice; see Section 4). This treatment resulted in a significant reduction of the basal Twitch-2B ratios of individual glomeruli, accompanied by a decrease in the apparent map inhomogeneity. It also strongly inhibited the odor-evoked glomerular signals (Figure S4). The basal Twitch-2B ratios of individual glomeruli and the apparent map inhomogeneity largely recovered 24 h after treatment (Figure 2A–C). The application of 10 µL PBS alone also reduced basal Twitch-2B ratios, albeit to a smaller extent (median effect size 11.96 ± 11.75, n = 33 glomeruli, 3 mice), likely due to changes in airflow through the nose (see the description of Figure 3 below).

As the data described above likely only reflected a partial blockade of the OSN Na^+^ channels (due to blocker release far from the OSNs and/or its impeded penetration through the mucus), in the next series of experiments, we chemically ablated OSNs using olfactotoxin dichlobenil, which effectively destroys OSNs projecting to the dorsal olfactory bulb [22]. By 6 h after the i.p. injection of dichlobenil, we observed a visible reduction in apparent map inhomogeneity, with a strong and significant blockade of glomerular activity seen between 12 and 48 h after drug application (Figure 2D–F). Together, these data identify action-potential-driven OSN activity as a contributor to the glomerular map inhomogeneity in the resting state.

The action potential (AP) firing in OSNs can occur spontaneously, in a cell-autonomous fashion, or be driven by ambient odorants or airflow. To distinguish between these possibilities, we first reversibly plugged one nostril with the silicone elastomer Kwik-Cast (Figure 3A–D, see Section 4). Because, in mice, odors are inhaled through the nostrils into two segregated nasal passages [23,24], some 40–80 min after nostril plugging, the ipsilateral hemibulbs showed a significant and reversible reduction in the basal Twitch-2B ratios (normalized to control median (per mouse) values: 0.78 ± 0.1, n = 6 mice). Despite the reduction, none of the glomeruli became silent (basal Twitch-2B ratios of all glomeruli ≥ 2, Figure 3B), likely due to the endogenous firing of OSNs. The basal Twitch-2B ratios of glomeruli in contralateral hemibulbs did not change (Figure 3C). Similar results were obtained when calculating the effect size of the nostril blockade (Figure 3D).

To estimate the contribution of ambient odorants to map inhomogeneity, we first established the level of intrinsic variability by monitoring the basal glomerular Twitch-2B ratios for 30 min in room air, switched for 30 s to clean air applied from an air tank and devoid of ambient odorants, and subsequently added a small concentration of the odorant to the clean air (0.18% of saturated vapor (s.v.), 30-minute-long application; Figure 3E,F). In contrast to the nostril blockade (Figure 3D), the effect sizes of all aforementioned manipulations did not differ from 0 and were similar to the effect size of room air (Figure 3F).

We noticed, however, that some glomeruli (e.g., the one marked by an asterisk in Figure 3E) either slightly increased or decreased their Twitch-2B ratios during the aforementioned manipulations, reflecting heterogeneous changes that were masked at the group level. To capture these subtle but meaningful variations, we reanalyzed our dataset using the absolute ratio changes (ΔR). The fluctuations of the basal glomerular Twitch-2B ratios were negligible in room air. The ratios did not change significantly in clean air but did show a small but significant change during the long odorant application (Figure S5). This suggests a small but measurable contribution of ambient odorants to the glomerular map inhomogeneity in the resting state.

**Figure 4:**
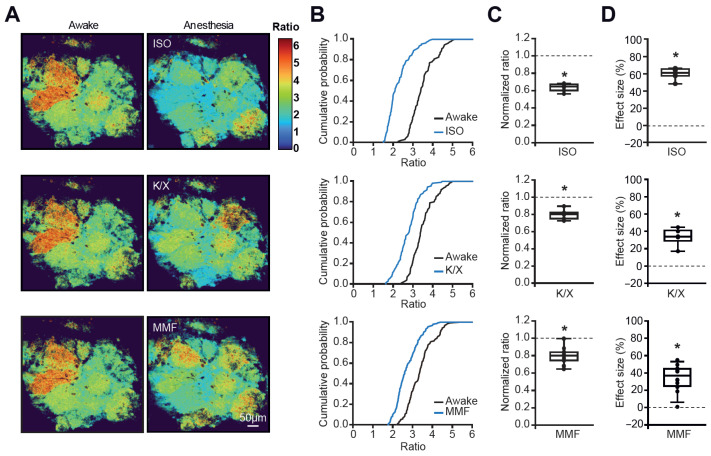
**Modulation of activity maps by anesthesia.** (**A**) Color maps, acquired pairwise (awake and anesthesia) from the same animals every second day and illustrating the effect of isoflurane (ISO, top), ketamine/xylazine (K/X, middle), and medetomidine/midazolam/fentanyl (MMF, bottom) on the basal Twitch-2B ratios. (**B**) Cumulative probability plots showing distributions of basal ratios under one of the 3 anesthetic regimens (ISO, K/X, MMF—blue) or the respective awake state (black). ISO and K/X: n = 110 glomeruli, 6 mice; MMF: n = 152 glomeruli, 12 mice. (**C**,**D**) Box plots illustrating the median (per mouse) normalized ratios (**C**) and effect sizes (**D**) for the 3 different types of anesthesia (one-sample *t*-test, ISO: *p* = 6.3 × 10^−6^ and 3.8 × 10^−6^; K/X: *p* = 4.2 × 10^−4^ and 3.1 × 10^−4^; MMF: *p* = 2.7 × 10^−6^ and 7.6 × 10^−6^, respectively). * *p* < 0.05, n.s = not significant.

To estimate whether the centrifugal inputs arising from the subcortical nuclei [25,26] might contribute to the glomerular activity map, we first examined the effects of different anesthetics (Figure 4A–D), which are collectively known to modulate the ascending reticular activating system (ARAS) [27,28]. The experiments were conducted every other day, with only one anesthesia regimen tested per day and corresponding awake recordings taking place immediately before anesthesia induction. All anesthesia regimens tested (isoflurane, ketamine/xylazine, or MMF (medetomidine, midazolam, fentanyl)) significantly blocked the glomerular activity (Figure 4A–D). The strongest effect was caused by isoflurane (median effect size: 62.25 ± 4%, n = 6 mice). We noted, however, that a small fraction of glomeruli (10% for isoflurane or ketamine/xylazine, 15.8% for MMF) increased their Twitch-2B ratios under anesthesia (Figure 4A).

**Figure 5:**
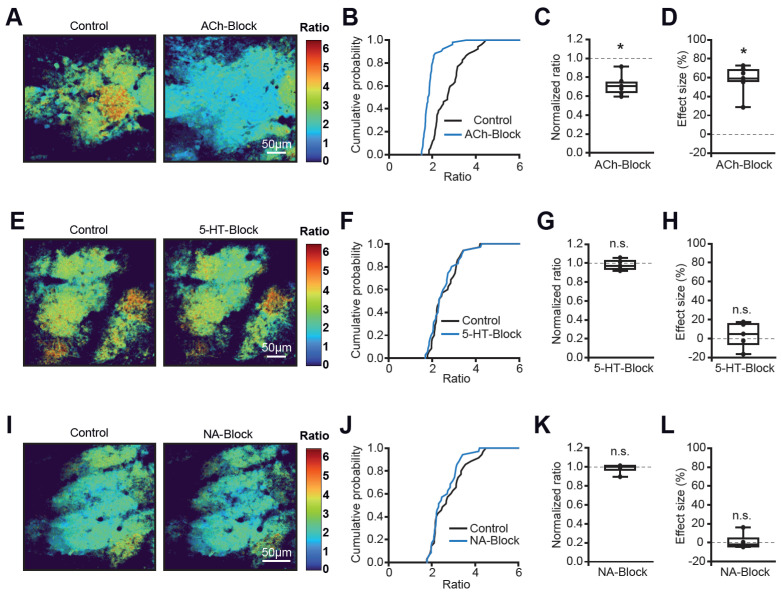
**Modulation of activity maps by centrifugal inputs.** (**A**,**E**,**I**) Color maps illustrating basal Twitch-2B ratios during topical application of HEPES-buffered Ringer solution in the absence (Control) and presence of cholinergic receptor blockers mecamylamine and scopolamine ((**A**), ACh-Block); serotonergic receptor blocker methysergide ((**E**), 5-HT Block); or α1-adrenergic receptor blocker prazosin ((**I**), NA-Block). (**B**,**F**,**J**) Cumulative probability plots showing distributions of basal Twitch-2B ratios in control and during one of the following pharmacological treatments: (**B**) mecamylamine (115 µM)/scopolamine (50 µM), n = 52 glomeruli, 7 mice; (**F**) methysergide (4 mM), n = 35 glomeruli, 5 mice; (**J**) prazosin (100 µM), n = 35 glomeruli, 5 mice. (**C**,**G**,**K**) Box plots showing the median (per mouse) normalized ratio values for the 3 pharmacological treatments. One-sample *t*-tests: (**C**) *p* = 3.13 × 10^−4^, n = 7 mice; (**G**) *p* = 0.55, n = 5 mice. (**K**) Wilcoxon Signed-Rank test: *p* = 0.81, n = 5 mice. (**D**,**H**,**L**) Box plots showing the respective median (per mouse) effect sizes. One-sample *t*-tests: (**D**) *p* = 4.1 × 10^−5^, n = 7 mice; (**H**) *p* = 0.59, n = 5 mice. (**L**) Wilcoxon Signed-Rank test: *p* = 0.81, n = 5 mice. * *p* < 0.05, n.s = not significant.

Because anesthesia, in addition to modulating ARAS, also affects respiration, we next tested the specific contributions of cholinergic, noradrenergic, and serotonergic projections by applying saturating doses of the respective receptor channel blockers onto the cranial window with a slit (Figure 5).

The application of the nicotinergic and muscarinergic receptor blockers mecamylamine (115 µM) and scopolamine (50 µM) strongly and significantly inhibited glomerular activity (Figure 5A–D), with 50% of glomeruli becoming silent (basal Twitch-2B ratios < 1.8), whereas the blockers of serotonergic (5-HT, 4 mM methysergide, Figure 5E–H) and noradrenergic (NA, 100 µM prazosin, Figure 5I–L) receptors had no effect. Together, these experiments identify cholinergic projections from the basal forebrain [26] as a strong contributor to the map inhomogeneity in the glomerular layer of the main olfactory bulb.

## 3. Discussion

Here, we directly visualize the glomerular activity map in awake head-restrained mice and show its striking inter-glomerular inhomogeneity. Within a given glomerulus, however, the activity levels were rather similar, underscoring the “functional unit” properties of glomeruli, previously seen when analyzing their odor-evoked responses [18,29]. Under resting awake conditions, the glomerular activity maps were impressively stable over days and weeks, but changed considerably under different types of anesthesia and with blockade of cholinergic centrifugal inputs, suggesting that they are brain-state-dependent. According to our data, the glomerulus-specific activity levels were sustained by the combination of (i) the ongoing endogenous spiking of olfactory sensory neurons, (ii) air-flow-stimulated OSN firing, (iii) modulatory cholinergic projections to the olfactory bulb, and, to a small extent, (iv) the ambient odor environment. Importantly, none of the aforementioned mechanisms alone could sustain the observed levels of inhomogeneity. The deciphered molecular mechanisms underlying the glomerular map inhomogeneity align well with previous data obtained in various preparations. Dissociated OSNs—OSNs in the isolated olfactory epithelium or olfactory epithelium slices, for example—are known to be endogenously active with spiking frequencies homogeneously distributed between 0 and 10 Hz [12,13,14,16,30]. Moreover, cells expressing different odorant receptor types are broadly tuned to distinct spiking frequencies and exhibit varying degrees of mechanosensitivity [13,16,30,31].

When stimulated by the patterned (artificial sniffing) or un-patterned airflow in anesthetized/awake head-fixed rodents, OSN axons showed airflow-induced responses in ~50% of all glomeruli [32,33]. The amplitudes of these airflow-driven responses varied between individual glomeruli, suggesting that the extent of mechanosensitivity is olfactory-receptor-specific. Interestingly, in the same preparation [33], the dendrites of airflow-responsive mitral cells projected to 80% of all glomeruli, hinting at mechanisms that enhance airflow-induced responsiveness beyond the input layer of the OB. The OSN inputs can also be further modified by the intrinsically bursting external tufted cells, which are entrained by these inputs and synapse on excitatory mitral cells, external tufted cells, and superficial tufted cells, as well as inhibitory periglomerular neurons [34,35].

The literature concerning cholinergic modulation in the OB, often stemming from electrophysiological recording in anesthetized preparations, remains inconclusive so far. Thus, electrical or optogenetic stimulation of the cholinergic neurons in the horizontal limb of the diagonal band of Broca, the main source of the cholinergic projections to the OB [36,37], caused the inhibition [38] or excitation [36,39] of the output mitral/tufted cells as well as the inhibition of glomerular and granule cell layer interneurons [38]. The stimulation also modulated the odor responses of individual glomeruli in different directions [36]. Moreover, the opposite effects were exerted by different subtypes of acetylcholine receptors (out of seven subtypes of nicotinic and two types of muscarinic acetylcholine receptors detected in the OB), with nicotinic receptors activating mitral, periglomerular and external tufted cells, and muscarinic receptors mediating either granule cell excitation (M1 receptors), which increases GABA release from granule cells onto mitral cells, or granule cell inhibition (M2 receptors) [36,37,40]. However, when only cholinergic axons projecting to the OB are optogenetically excited and not all cholinergic neurons in the diagonal band of Broca, the spontaneous firing of mitral cells as well as their responses to the artificial inhalation of clean air were enhanced [41], well in line with a strong reduction in the glomerular map activity observed under cholinergic receptor blockers in our study (Figure 5). In fact, the latter treatment completely silenced 50% of all glomeruli, consistent with the dense cholinergic innervation of both glomerular and mitral cell layers of rodents and primates [39,41,42].

Our data unify all of the scattered information described above by introducing and directly visualizing an extremely stable, inhomogeneous map of basal glomerular activity as the background against which olfactory signal processing occurs. The described basal glomerular activity map adds another layer to the complex computational network of the OB, increasing the dynamic range and variance of the system via glomerulus-specific functional inhomogeneity. Moreover, it enables a response to odorants not only with graded increases (ON-glomeruli), but also with graded decreases (Inhibited-glomeruli, [18]) in glomerular activity. The importance of this new layer for signal processing is emphasized by computational/machine learning studies suggesting that functional inhomogeneity in neural networks allows for an increase in computational power and performance at low cost (metabolic efficiency), and also promotes and stabilizes robust learning (computational efficiency), improves generalization, and enhances pattern separation [43,44,45].

## 4. Materials and Methods

### 4.1. Animals

In this study, we used adult *C57BL/6N* wildtype (WT) mice of either sex, bred in our animal facility. Female mice were housed in groups of 3–5, while male mice were conventionally housed in individual neighboring cages with all olfactory, visual, and acoustic stimuli preserved, to avoid fights and injuries. Unless otherwise indicated, all animals were kept under a 12 h light/dark cycle with ad libitum access to food and water.

### 4.2. Ethics Statement

All experimental procedures complied with the ARRIVE guidelines and were carried out in accordance with the EU Directive 2010/63/EU on animal experiments. The animal study protocol was approved by the state government of Baden-Württemberg, Germany (protocol code 35/9185.83-2 from 16 June 2017).

### 4.3. Labeling of the Olfactory Bulb with the Ca^2+^ Indicator Twitch-2B

To label neural networks in the olfactory bulb, an AAV-based viral vector encoding the FRET-based Ca^2+^ indicator Twitch-2B (Kd = 200 nM) under the human Synapsin1 promoter (AAV1-hSyn1-Twitch-2B, Addgene viral prep # 100040-AAV1, Boston, MA, USA) was used [46]. The virus was diluted 1:7, and 1 µL of the virus-containing solution was stereotactically injected mediocaudally (at 0.35 mm, 0.25 mm, and 0.15 mm depth (~300 nl per injection) and a 45° angle from the horizontal plane) into both olfactory hemibulbs of 2- to 6-month-old mice, followed by chronic cranial window implantation (see below). This enabled the labeling of OSN axons (Figure S6) as well as periglomerular [20] and mitral/tufted cells (Figure S7 in [19]) with Twitch-2B.

Compared to single-wavelength Ca^2+^ indicators like GCaMPs, the use of ratiometric FRET-based indicators, including Twitch-2B, provides more accurate measurements better suited for long-term in vivo functional imaging studies because they are less influenced by changes in the optical path length, excitation light intensity, indicator expression level, and movement artefacts. Moreover, Twitch-2B is substantially brighter than GCaMPs in non-spiking neurons, allowing for the better identification of expressing cells and their subcellular structures. It also has better linearity as well as reduced Ca^2+^ buffering capacity and cytotoxicity due to a reduced number of Ca^2+^ binding sites (two in Twitch-2B vs. four in GCaMPs; [46]).

### 4.4. Chronic Cranial Window Implantation

Surgery was performed as described earlier [20,47]. Briefly, the mice were anesthetized with an intraperitoneal (i.p.) injection of midazolam (5 mg/kg BW), medetomidine (0.5 mg/kg BW), and fentanyl (0.05 mg/kg BW), hereafter abbreviated as “MMF” anesthesia. The surgical depth of the anesthesia was confirmed by the absence of the toe pinch reflex. Before surgery, an eye ointment (Bepanthen, Bayer, Leverkusen, Germany) was applied to prevent the cornea from drying out. Then, xylocaine (2% *w*/*v*) was injected subcutaneously (s.c.) to induce local anesthesia at the incision site, and dexamethasone (0.2 mg/kg BW, Sigma-Aldrich Merck, Darmstadt, Germany) was injected s.c. to prevent swelling of the brain upon removal of the bone. Body temperature was monitored with a rectal temperature probe and maintained between 36 and 37 °C. After removing the skin above the OB, two craniotomies were made for each olfactory hemibulb using a dental drill (NSK, Ultimate 500, NSK Europe GmbH, Eschborn, Germany): the region around both OB hemispheres and the midline bone covering the olfactory sinus between the hemispheres was thinned until two loosely attached islands formed. Subsequently, Ringer’s injection solution (B. Braun, Melsungen AG, Pfieffewiesen, Germany) was applied to prevent the brain surface from drying out, and the islands were gently removed with forceps while the midline bone above the olfactory sinus, as well as the dura, were left intact. Both hemibulbs were covered with one round coverslip (Ø 3 mm, Warner Instruments, Hamden, CT, USA). The remaining exposed skull was covered with dental cement (Tetric EvoFlow, Ivoclar Vivadent GmbH, Ellwangen, Germany), and a small stainless steel or titanium holder was fixed to the skull caudally to the coverslip. Postoperative care included an analgesic dose of carprofen (5 mg/kg BW, Pfizer Pharma GmbH, Berlin, Germany) for 3 days s.c. and 0.025% of the antibiotic enrofloxacin (Baytril, Bayer AG, Leverkusen, Germany) in drinking water for 10 days. The two-photon imaging commenced 3–4 weeks after the surgery. Before the first awake two-photon imaging session, the mice were habituated to fixation in the imaging setup for 10–15 days by daily fixations, lasting between a few seconds (at the beginning of training) and up to 90 min (at the end of training).

To apply receptor antagonists to the surface of the olfactory bulb, trained mice were implanted with a chronic cranial window as detailed above, and the coverslip contained a 1.0 × 0.1 mm slit [21], located rostrally above the cavity between the OB hemispheres. At the end of the surgery, the slit was covered with Kwik-Cast and sealed on top with Kwik-Sil (both are silicone elastomers from World Precision Instruments Germany GmbH, Berlin, Germany), and imaging experiments commenced one day later. The silicon elastomers were removed before the drug application.

### 4.5. In Vivo Two-Photon Calcium Imaging

Only trained mice, habituated to the setup and the head fixation, were used. In awake mice, the respiration rate was monitored using a thermistor (Murata NCP15XH103J03RC; Conrad Electronic SE, Hirschau, Germany) positioned in front of one nostril. In anesthetized mice, the respiratory rate was monitored using a pressure sensor (ADInstruments, Dunedin, New Zealand) attached to the back of each mouse. Under isoflurane anesthesia, the isoflurane concentration was maintained at 0.9–1.5% in O_2_ to keep the respiratory rate between 110 and 160 breaths per minute. Otherwise, mice were injected i.p. with ketamine/xylazine (80/4 µg per g BW) or MMF (composition see above). The animals breathed freely throughout the experiments. Two-photon imaging started 10–15 min after anesthesia induction.

We used an Olympus Fluoview 1000 laser scanning microscope (Olympus Europa SE & Co. KG, Hamburg, Germany) coupled to a mode-locked Ti:Sapphire laser (Mai Tai DeepSee, Spectra Physics, High Q Laser GmbH, Rankweil, Austria) and operating at 690–1040 nm with a pulse width of <100 fs and a repetition rate of 80 MHz. The regions of interest to be imaged were chosen in areas with the best clarity of the cranial window (i.e., devoid of bone regrowth and large brain vessels). The highest number of glomeruli in one plane of focus, and in different mice, were located in the front (n = 6), middle (n = 17) and back (n = 15) of the implanted cranial window. Images were acquired using an excitation wavelength of 890 nm and a 20× Plan-Apochromat 1.0 NA water-immersion objective (Carl Zeiss AG, Oberkochen, Germany). Emitted light was split using a 515 nm dichroic mirror and a 475/64 nm bandpass or a 500 nm long-pass filter for mCerulean3 and cpVenus^CD^ channels, respectively. For time series, three consecutive sequences of 80 (Figure 5) or 1000 (Figure S3) frames at a rate of 4–7.8 Hz with an inter-sequence interval of 30 to 60 s, and an image size of 512 × 256 pixels, were recorded at a single depth level within the glomerular layer. For the three-dimensional reconstruction of the glomerular layer, images were acquired from the dura mater to a depth of approximately 120 μm with an image size of 640 × 640 pixels, using a Kalman filter setting of 3 and a step size of 2 µm.

### 4.6. Odorant Delivery

In most experiments, glomerular basal Twitch-2B ratios were measured in mice that were freely breathing ambient air in a ventilated room, termed “Room air”. In some experiments, the room air was replaced by “Clean air” from an air tank, by placing a custom-built flow-dilution olfactometer (see Figure 3.8 in ref. [48]) approximately one centimeter in front of the mouse’s snout and providing a constant clean airflow at the rate of 300 mL/min. To record odor-evoked Ca^2+^ signals (Figure S4), ethyl tiglate (ETI), ethyl butyrate (EBU), or isoamyl acetate (IAA), all known to activate the dorsal OB [49], were applied via the flow-dilution olfactometer, in which the pure air was mixed with the saturated odorant vapor. For continuous odor application over 30 min, the odorant concentration of 0.18% s.v. (“Clear air + long odor on”) was used. All odorants were purchased from Sigma-Aldrich Merck at the highest commercially available purity.

### 4.7. Local Application of Antagonists in Awake Mice

As inflammation-induced changes in the tissue function were reported to begin 2 days after surgery [50,51], and because the dura mater in our preparations became impermeable to drugs after 4 weeks (unpublished observation), we applied receptor antagonists 12–24 h after surgery. Drugs (all from Sigma-Aldrich Merck) were diluted in HEPES-buffered Ringer solution (in mM: 150 NaCl, 4.5 KCl, 10 HEPES, 1MgCl_2_, 1.6 CaCl_2_, pH 7.4). A drop of around 40 µL was applied onto the cover glass with a slit. The following drugs/concentrations were used: the voltage-gated Na^+^ channel blocker tetrodotoxin (TTX, 5 µM), the nonselective α1- and α2-adrenergic receptor antagonist prazosin (100 µM), the nonselective 5-HT_1_-, 5-HT_2_-, and the 5-HT_7_-serotonin receptor antagonist methysergide (4 mM). The mAChR-antagonist scopolamine and the nAChR-antagonist mecamylamine were prepared first in HEPES-buffered Ringer solution and then mixed to a final concentration of 50 mM and 115 mM, respectively. To ensure that sufficient drug concentrations reached the target cells via diffusion through the small slit, we used higher concentrations than those previously described in anesthetized mice when perfusing a wide area of the OB surface [25,41]. The initial imaging session was performed with the Kwik-Cast/Sil still covering the slit (see ref. [20] for further details). Then, the Kwik-Cast/Sil was gently removed to apply the HEPES-buffered Ringer solution (Figure 5, Control) and, subsequently, the receptor blockers (Figure 5, Block).

### 4.8. Modulating the Activity of Olfactory Sensory Neurons

To address the contribution of olfactory sensory neurons (OSNs) to the overall basal glomerular activity levels, two-photon imaging experiments were performed in awake animals. Only trained mice, habituated to the setup and the head fixation routine, were used. After recording basal glomerular Twitch-2B ratios (Control) and odor-induced responses (Figure S4, details see below), the mice were returned to their home cage for five minutes. Thereafter, the OSNs were targeted in one of three different ways, as described below.

*Non-invasive intranasal application of TTX:* To block OSN activity within the sensory epithelium, a non-invasive intranasal application of TTX (50 µM in PBS) was used. Here, 10 µL of the TTX-containing solution was applied twice as droplets directly into one nostril of the mice, who were briefly (≤2 min) anesthetized with isoflurane. The mice were returned to their home cages and allowed to rest for 10 min to recover from the anesthesia. Thereafter, a second identical set of TTX applications was performed in the awake state. Immediately after application of the blocker, the mice were head-fixed in the setup, and the same region of interest as under control conditions was imaged.

*Dichlobenil administration:* For the chemical ablation of olfactory sensory neurons, the known olfactotoxic substance dichlobenil (2,6-Dichlorobenzonitrile, [22]) was administered via a single i.p. injection (50 µg/g body weight in 1 µL/g body weight DMSO) after the control imaging session. The effect of dichlobenil was monitored over time, with imaging sessions at 3, 6, 9, 12, 24, and 48 h after dichlobenil administration. Between the imaging sessions, the mice were transferred to their home cage.

*Nose plugging:* For blocking the activity of OSNs caused by the air-flow-induced mechanosensation and ambient odors, one nostril was reversibly plugged by the silicon elastomer Kwik-Cast under short (5–8 min) isoflurane anesthesia. To do so, the mouse was placed on its back on a heating pad, and the two-component liquid elastomer was carefully applied to the nostril. The anesthesia was maintained, and the viscose elastomer was allowed to harden, thus forming a tight-fitting plug. Immediately thereafter, the isoflurane supply was interrupted, and the mouse was transferred to the setup to be imaged at different time points (Figure 3). We used a plastic collar to prevent the awake animal from removing the plug. After the measurement, the collar was removed, allowing the mice to remove the plug within 1–2 min. After reopening the nostril, the imaging session resumed.

### 4.9. Immunohistochemistry

At the end of the experiment, the olfactory bulbs were isolated and fixed with 4% formaldehyde in PBS for 24 h at 4 °C and cryoprotected in 25% sucrose in PBS overnight at 4 °C. Afterward, they were embedded in Tissue Tek (Sakura Finetek, Torrance, CA, USA) and stored at −80 °C until use. The free-floating OB slices were treated with a blocking solution (5% normal donkey serum and 1% Triton-X in PBS) for 1 h before overnight incubation at 4 °C with either the primary goat-anti-OMP (Olfactory marker protein; 1:1000, Wako, TX, USA, 544-10001) or rabbit-anti-GFP recognizing Twitch-2B (1:1500, Rockland, ME, USA, 600-401-215) antibodies, diluted in blocking solution. After rinsing the slices 3 × 10 min in PBS, the slices were incubated in the dark in a 2% BSA solution containing Alexa Fluor 488- and Alexa Fluor 594-conjugated secondary antibodies (1:1000 dilution for both) for 2 h at room temperature. Thereafter, the slices were washed 3 × 10 min in PBS in the dark and mounted on fluorescence-free Superfrost microscope glass slides in Vectashield mounting medium.

Immunohistochemically labeled OB slices were imaged using the Olympus Fluoview 1000 laser scanning microscope described above (Section 4.5) through a 40× water-immersion objective (0.80 NA, Nikon, Tokyo, Japan) at an excitation wavelength of 800 nm. A 585 nm dichroic mirror was used to split the light emitted by the two Alexa dyes. A 536/40 nm bandpass and a 585 nm long-pass filter were used to filter the Alexa Fluor 488 and the Alexa Fluor 594 emission light, respectively.

### 4.10. Data Analyses

*Time series of basal and odor-evoked Ca^2+^ signals:* For the analysis of glomerular signals, regions of interest (ROIs) were drawn manually in Fiji (ImageJ 1.53c, Java 1.8.0_172 https://imagej.net/Fiji (accessed on 15 April 2026)). A reference background ROI was drawn in the darkest spot of the image. Further analyses were performed using custom-written scripts in MATLAB (Version: 25.1.0.2973910 (R2025a) Update 1, Image Processing Toolbox Version 25.1 (R2025a) and earlier, Natick, MA, USA: The MathWorks Inc. https://www.mathworks.com (accessed on 15 April 2026)). The respective mean background intensities were subtracted from the mean fluorescence intensities in the mCerulean3 and cpVenus^CD^ channels before cpVenus^CD^/mCerulean3 ratios were calculated for all ROIs. Odor-evoked Ca^2+^ transients were counted as responses when their ΔR/R signal was six times larger than the standard deviation of the corresponding baseline noise, and when a minimum of 15% ΔR/R was reached.

*Three-dimensional measurements of basal glomerular Twitch-2B ratios:* We used a custom-written MATLAB script to load 3D stacks, draw eight ROIs for each glomerulus at different levels that cover the entire glomerular volume, and extract mean fluorescence intensity values for mCerulean3 and cpVenus^CD^. The background fluorescence was subtracted individually for each channel and z-level, and the cpVenusCD/mCerulean3 ratio was calculated. The resulting median ratio values for the eight z-levels were taken as the basal ratio for an individual glomerulus.

*Computation of color-coded ratio maps (color maps):* To compute the color-coded ratio maps, each full-frame image (640 × 640 pixels) of the gray-scale 3D image stack was filtered with a 7 × 7 average filter. Then, the data were analyzed as described above, but ratios were calculated per pixel for pixels in the image with mean intensities between 100 and 2500. The remaining pixels were blanked to avoid over- (when dividing by a small number) or underestimation (when including the saturated pixels of cell somata) of ratio values. The resulting ratio images were color-coded with a colorblind-friendly continuous color map (turbo; https://research.google/blog/turbo-an-improved-rainbow-colormap-for-visualization/ (accessed on 15 April 2026)).

*Calculation of the effect size:* In the experiments shown in Figure 2, Figure 3, Figure 4 and Figure 5, the reduction in the basal Twitch-2B ratio was calculated as a percentage of the maximal block achievable, termed “effect size”. The maximum theoretically possible block was defined as the reduction to the lowest glomerular ratio level (1.3) ever measured under the TTX application to the surface of the olfactory bulb (Figure S2). We calculated the difference between the basal ratio measured in the control condition and 1.3 (R_ctr_ − 1.3) and the difference between the basal ratios measured in the control and test conditions (R_ctr_ − R_test_). The effect size (in %) was then calculated according to the formula

$$effect\;size\left(\%\right)=\frac{R_{ctr}-R_{test}}{R_{ctr}-1.3}\times 100$$

*Statistical analysis and data presentation:* Statistical tests were performed in MATLAB, GraphPad Prism (Version 9.5.1, www.graphpad.com (accessed on 15 April 2026)), Igor Pro (Version 6.3.7.2, WaveMetrics, Portland, OR, USA), R version 4.2.1 (https://cran.uni-muenster.de/ (accessed on 15 April 2026)), or Vassar Stats (website for statistical computation, http://vassarstats.net/ (accessed on 15 April 2026)). The one-sample Kolmogorov–Smirnov test was used to assess the normality of the data distribution. The Levene test was used to check for homoscedasticity. Non-parametric tests were performed when the assumptions of parametric tests were violated. In cases with a repeated-measure design and ANOVA assumption violations (Figure 1F, Figure 2C,F and Figure 3D), we performed the Friedman test followed by the Conover post hoc test and Bonferroni correction, considering the control values as a separate group. All statistical tests were two-sided. *p*-values smaller than 0.05 were considered significant. Unless otherwise indicated, data are presented as median ± interquartile range. Lines in box plots represent the 25th and 75th percentiles, and whiskers show the minimum and maximum data points, respectively. All bar, box, cumulative, or x-y scatter plots were calculated and plotted with custom-written MATLAB scripts and displayed with Adobe Illustrator CC 2017 (Adobe Systems Software Ireland Limited, Dublin, Republic of Ireland).

## Data Availability

The original contributions presented in this study are included in the article/Supplementary Material. Further inquiries can be directed to the corresponding author.

## Supplementary Materials

The following supporting information can be downloaded at: https://www.mdpi.com/article/10.3390/ijms27083684/s1.

## Abbreviations

The following abbreviations are used in this manuscript:

OB	Olfactory bulb
MMF	Medetomidine, Midazolam, Fentanyl
K/X	Ketamine/Xylazine
TTX	Tetrodotoxin
OSN	Olfactory sensory neurons
OR	Odor receptor
M/T	Mitral/tufted
ROIs	Regions of interest
FRET	Förster resonance energy transfer
ARAS	Ascending Reticular Activating System
